# Metformin-Associated Lactic Acidosis in an Older Adult: A Case Report and Review

**DOI:** 10.7759/cureus.62729

**Published:** 2024-06-19

**Authors:** Sakshi Jain, Sonia Sekhon, Angelin Shamili Leo Pradeep Chandran, Jyotsna Gummadi, Premalkumar Patel, Raghuma Nakka, Tharajan Gunendran, Athmananda Nanjundappa, Tom Jose, Hari Naga Garapati, Saketh Palasamudram Shekar, Amaraja Kanitkar

**Affiliations:** 1 Geriatrics, Hackensack University Medical Center, Hackensack, USA; 2 Internal Medicine, Baylor St. Luke's Medical Center, Houston, USA; 3 Paediatrics, Tulane Lakeside Hospital, Metairie, USA; 4 Medicine, MedStar Franklin Square Medical Center, Baltimore, USA; 5 Infectious Disease, Mount Sinai Medical Center, Miami Beach, USA; 6 Internal Medicine, Jackson Park Hospital and Medical Center, Chicago, USA; 7 Internal Medicine, All Saints University, Roseau, DMA; 8 Internal Medicine, MedStar Franklin Square Medical Center, Baltimore, USA; 9 Internal Medicine, University of North Carolina (UNC) Lenoir Hospital, Kinston, USA; 10 Montgomery Kidney Specialists, Jackson Hospital, Montgomery, USA; 11 Pulmonary and Critical Care Medicine, Pulmonary and Sleep Associates of Huntsville, Huntsville, USA; 12 Pulmonary and Critical Care Medicine, Ascension Macomb-Oakland Hospital, Warren, USA

**Keywords:** metformin-associated lactic acidosis, chronic kidney disease, aging kidney, egfr, lactic acidosis, acute kidney injury, older adult, elderly, renal failure, metformin

## Abstract

Metformin is a widely prescribed medication for the management of type 2 diabetes. It is known to have a high safety index; however, it can cause serious adverse effects such as lactic acidosis, particularly in patients with chronic kidney disease. Elderly patients are at higher risk of developing metformin-associated lactic acidosis (MALA) due to aging kidneys. We present an 82-year-old male with a past medical history of diabetes, stage 2 chronic kidney disease, atrial fibrillation on apixaban, stroke, and chronic stage 4 sacral decubitus ulcer who was sent to the emergency department (ED) for altered mental status. He was admitted to the intensive care unit for the management of septic shock, pulseless electrical activity (PEA) cardiac arrest, and acute hypoxemic respiratory failure requiring intubation. Laboratory tests showed lactic acidosis and anion gap metabolic acidosis in the absence of an infectious source. The patient had chronic kidney disease with acute renal failure on metformin. He was diagnosed with MALA. This case highlights the potential risks associated with metformin use in older adults with chronic kidney disease and acute kidney injury from infections, dehydration, and decreasing oral intake due to acute illness, aging, or dementia. There are expected physiological changes in the aging kidney, including cellular dysfunction and nephrosclerosis, that can cause unexpected kidney injury in older adults, causing their estimated glomerular filtration rate (eGFR) to drop acutely. Age-related changes in renal function and decreased clearance of drugs place elderly patients at higher risk of developing MALA. Guidelines for reducing or deprescribing metformin can be considered in older adults. This could prevent morbidity, mortality, and adverse outcomes in frail older adults with diabetes.

## Introduction

Metformin has emerged as one of the most widely prescribed oral hypoglycemic agents for managing type 2 diabetes mellitus. The use of biguanides, such as metformin, has shown a significant increase over the years, surging from 8.3% in 1998 to 54.3% in 2010 [1]. Research has demonstrated that metformin can reduce the postprandial glycemic index by 20-30% and can prevent the occurrence of both small and large blood vessel diseases [2-5]. Additionally, it leads to a substantial decrease in overall mortality of 36%, as well as a 32% decrease in diabetes-related complications and a remarkable 47% decline in diabetes-related deaths [6]. Furthermore, metformin has shown various other impacts, including the amelioration of non-alcoholic steatohepatitis [7]. Metformin's effectiveness in improving blood sugar control, affordability, and favorable side effect profile have made it popular among healthcare professionals and patients alike. Despite the numerous benefits it offers, certain vulnerable groups, particularly individuals with renal disease and reduced estimated glomerular filtration rates (eGFR) less than 45 ml/min, may experience tissue accumulation of metformin. This accumulation can lead to a potentially life-threatening complication known as metformin-associated lactic acidosis (MALA) [8]. 

We present a case report of an older adult with diabetes, chronic kidney disease (CKD), and a stage 4 decubitus ulcer who experienced an acute episode of altered mental status. Further investigation revealed that the underlying cause of the anion gap metabolic acidosis was renal failure with metformin toxicity contributing to the development of lactic acidosis. There are expected physiological changes in the aging kidney, including cellular dysfunction and nephrosclerosis that can cause unexpected kidney injury in older adults [9]. This can result in a sudden drop in eGFR and a longer recovery time in older adults compared to younger adults. Guidelines for early reduction or deprescribing metformin can be considered, which could prevent morbidity and mortality in frail older adults with diabetes.

This article was previously presented as a meeting abstract at the 2023 American Medical Association research challenge on October 18, 2023. 

## Case presentation

An 82-year-old male with a past medical history of diabetes, stage 2 CKD, atrial fibrillation on apixaban, stroke, and chronic stage 4 sacral decubitus ulcer was sent to the emergency department (ED) for altered mental status and admitted for bacteremia due to sacral ulcer. He was started on oral amoxicillin/clavulanic acid for six months and was discharged. At the rehab nursing facility, he was noticed to have poor oral intake, low blood glucose levels ranging from 80 to 90 mg/dL (normal 70-130 mg/dL), and eGFR of 43 (normal >60), so his long-acting sulfonylurea was discontinued and metformin was decreased from 1000 mg twice a day to 500 mg twice a day. Glucose levels remained within an acceptable range between 100 and 150 mg/dL for his eGFR.

Three months later, he was readmitted from the nursing facility for an abnormally elevated white blood cell (WBC) count of 22 K/UL (normal 4.5-11.0 microliter). The stool test was positive for *Norovirus*, enterotoxigenic *E. coli*, and *Clostridium difficile* infection. He was given intravenous fluids and oral vancomycin to finish the 10-day course. He was placed back on oral amoxicillin and clavulanic acid for his sacral ulcer and discharged back to the nursing facility.

One month later, the patient was noted to be lethargic. He was sent to the ED, where labs showed he was acidotic with a pH of 6.5 (normal 7.35-7.45) and a bicarbonate level of 2 (normal 22-29 mEq/L) and lactic acid levels were 15 (normal <2 mmol). His eGFR was found to be 18. More laboratory values can be seen in Table 1. He was admitted to the intensive care unit for the management of possible septic shock, pulseless electrical activity (PEA) cardiac arrest, and acute hypoxemic respiratory failure requiring intubation. Differentials included septic shock from an unclear source. He was empirically started on intravenous meropenem and vancomycin. The infectious source was initially thought to be from his known sacral decubitus ulcer although he had already been on oral antibiotics for this for a few months. He received dialysis for acute renal failure and acidosis. The patient was seen by infectious disease consultants, and his chronic sacral wound was deemed to be an unlikely source of infection as he was recently treated for it, he was on long-term oral antibiotics, and the wound appeared clean. Due to low suspicion for infection leading to septic shock and cardiac arrest, he was diagnosed with anion gap metabolic acidosis secondary to renal failure and MALA from metformin toxicity. A metformin level was not ordered as he had already received dialysis and his levels would not have been accurate. He was stabilized and discharged back to the nursing home. 

**Table 1:** Laboratory values BUN: blood urea nitrogen; eGFR: estimated glomerular filtration rate; PO2: partial pressure of oxygen; PCO2: partial pressure of carbon dioxide

Normal lab values	Rehab facility	First hospitalization	Second hospitalization
Potassium 3.3-5.1 mmol	4.3	4.5	5.7
Bicarbonate 20-32 mmol/L	22	20	2
BUN 7-22 mg/dL	32	34	51
Creatinine 0.6-1.4 mg/dL	2.61	2.79	4.12
eGFR >59 ml/min	43	40	18
Anion gap 4-16	12	13	21
Lactic acid 0.5-1.6 mm/L	-	2.3	15
HbA1C 4-6%	-	-	5.9
pH 7.35-7.45	-	-	6.5
PO2 80-100 mmHg	-	-	55
PCO2 35-45 mmHg	-	-	47

## Discussion

MALA is characterized by the buildup of lactate in the bloodstream due to impaired lactate clearance, leading to a decrease in pH and bicarbonate levels. This metabolic disturbance can result in severe clinical manifestations and pose significant risks to the patient's health and well-being [10]. It is crucial to promptly recognize and diagnose MALA, as delayed treatment can have grave consequences for those affected.

Table 2 summarizes case reports of MALA across different geographic regions of the world with patients of varying ethnicities and races.

**Table 2:** Previously published studies and case reports of MALA with demographics, initial presentation, diagnostic tests, findings, and management CBC: complete blood count; CMP: comprehensive metabolic panel; ABG: arterial blood gas; CPR: cardiopulmonary resuscitation; ECG: electrocardiogram; PEA: pulseless electrical arrest; ICU: intensive care unit; CKD: chronic kidney disease; ECMO: extracorporeal membrane oxygenation; LVEF: left ventricular ejection fraction; PO2: partial pressure of oxygen; PCO2: partial pressure of carbon dioxide; V-A ECMO: venoarterial extracorporeal membrane oxygenation

Study name	Demographics	Initial presentation	Diagnostic tests performed	Findings	Management/outcomes
Umeda et al. [11]	54-year-old Hispanic female	The patient presented with watery diarrhea, nausea, vomiting, and mental status changes	Blood glucose level, CBC, CMP, ABG, cardiac biomarkers, serial ECGs, echocardiogram, nuclear stress test, coronary angiography	Laboratory tests found a blood glucose level of 47 mg/dL, creatinine of 8.07 mg/dL, and elevated troponin of 4.42 ng/mL. ABG showed an arterial pH of 6.57 and bicarbonate of 2 mEq/L. Cardiac workup: Serial ECGs were negative for ST-T wave changes. Echocardiogram revealed a preserved ejection fraction of 50%. Nuclear stress test was abnormal. Coronary angiography revealed three vessel diseases with 80-90% stenosis	Management consisted of glucagon for hypoglycemia, CPR, endotracheal intubation, shock management, hemodialysis, and a coronary artery bypass graft. Outcome: The patient was discharged on day 15. Dialysis was discontinued in one month as renal function returned to baseline
Kinoshita et al. [12]	70-year-old Japanese female	The patient presented with diarrhea, nausea, and vomiting for three days with altered mental status	CBC, CMP, serum, metformin level, ABG	Laboratory tests found severe lactic acidosis with a pH of 6.618, bicarbonate of 1.7 mmol/L, lactate level of 18 mmol/L, severe acute kidney injury with BUN of 67.5 mg/dL and creatinine of 10.17 mg/dL, and metformin concentration of 77.5 mg/dL	Management consisted of intravenous fluids, sodium bicarbonate, antibiotics, vasopressors, endotracheal intubation, and continuous renal replacement therapy. Outcome: Discharged from hospital on day 20
Ncomanzi et al. [13]	66-year-old Caucasian female	The patient presented with a three-week history of generalized malaise, poor oral intake, and diarrhea	CBC, CMP, cardiac biomarkers, venous blood/gas measurement, serum metformin level	Labs showed sodium of 140 mmol/L, potassium of 7.3 mmol/L, bicarbonate of 1 mmol/L, BUN of 30.8 mmol/L, creatinine of 7.68 umol/L, anion gap of 55 mmol/L, troponin level of 50 ng/L, and metformin level of 4 mg/L. Venous blood gas measurements: profound metabolic acidemia: pH, 6.58; HCO_3_, 3.6 mmol/L; glucose, 2.0 mmol/L; and lactate, 16.7 mmol/L	Management consisted of endotracheal intubation and ventilation for PEA arrest, intravenous fluids, vasopressors, continuous renal replacement therapy, and intravenous antibiotics. Outcome: Discharged home on day 16 after a total 35-day stay in the ICU
Fadden et al. [4]	58-year-old female	The patient presented with diarrhea, vomiting, abdominal pain, and poor oral intake	CBC, CMP, lactate level, ABG	Labs showed a pH of 6.6, lactate level of 14 mmol/L, acute kidney injury, elevated WBC to 12.5×10^9^/L on CBC, and glucose of 2.5 mmol/L. CT of the abdomen showed possible pancolitis	Management consisted of supportive treatment for brief systolic cardiac arrest, renal replacement therapy, and intravenous dextrose for severe hypoglycemia. Outcome: Discharged home on day 16
Shenoy [5]	48-year-old male	The patient presented with nausea, vomiting, diarrhea, abdominal pain, and lethargy	CBC, CMP, lactic acid level, ABG	Labs showed WBC of 33,000/mm^3^, bicarbonate of 7.0 mEq/L, creatinine level elevated to 2.9 mg/dL, BUN of 16 mg/dL, anion gap of 33, lactic acid level of 25.0 mmol/L, ABG with pH of 6.85, PCO2 of 17, and PO2 of 133 with an oxygen saturation of 95% on room air. The chest X-ray and ECG were normal	Management included intravenous fluids for viral gastroenteritis, bicarbonate drip, hemodialysis, intubation, and ventilator support for respiratory distress. Outcome: Discharged home five days after admission on rosiglitazone
Ashraf et al. [6]	53-year-old African American female	The patient presented with dizziness, generalized weakness, fatigue, and shortness of breath	CBC, CMP, ABG	Labs showed blood sugar of 34 mg/dL, hemoglobin A1c of 6.9%, CKD stage 3a with eGFR 8.51 mL/minute, creatinine of 6.6 mg/dL, potassium of 6.7 mmol/L, ABG with severe anion gap metabolic acidosis, and lactate of 20 mmol/L	Management with dextrose for hypoglycemia, broad-spectrum intravenous antibiotics, and dialysis. Outcome: Discharged back to the nursing home on day 8
Plumb et al. [14]	66-year-old female	The patient presented with a five-day history of diarrhea, vomiting, and abdominal pain	ABG, CBC, CMP, ECG, bedside echocardiogram	Labs showed a potassium of 7.4 mmol/L. ABG showed a pH of 6.57. ECG: No p waves, irregular broad complexes, and tall T waves consistent with hyperkalemia. The echocardiogram showed myocardial infarction	Management of pulseless electrical activity cardiac arrest with vasopressors and hemodialysis and methylene blue infusion. Outcome: Discharged home on day 18
White et al. [15]	52-year-old female	The patient presented with altered mental status, hypoglycemia, and shock	Prehospital ECG, metformin level	Labs showed a metformin concentration of 51 g/mL. ABG showed severe metabolic acidosis. The ECG showed findings of posterolateral ST-elevation myocardial infarction	Management consisted of emergent hemodialysis and treatment for acidosis and hypothermia. Outcome: Discharged from hospital
Chen et al. [16]	72-year-old female	The patient presented with nausea and fatigue	ECG, ABG, echocardiography	ABG showed a pH of 6.80, lactic acid of >15 mmol/L, and PCO2 of 14 mmHg. ECG: tachycardia with a wide QRS. Echocardiography showed a diffuse reduction of left ventricular wall motion, and the LVEF was 23.5%	Management included sodium bicarbonate drip, vasopressors, IVFs, dobutamine drip, V-A ECMO, and intubation with mechanical ventilation. Outcome: Discharged from the hospital on day 10
Hai et al. [17]	66-year-old male	The patient presented with fatigue, abdominal pain, nausea, vomiting, and severe diarrhea for three days	CBC, CMP, ABG	ABG showed pH of 6.94, PO2 of 151 mmHg, PCO2 of 14 mmHg, bicarbonate of 3 mmol/L, anion gap of 48 mmol/L, and lactate level of 15 mmol/L	Management included sodium bicarbonate and glucose control with insulin infusion, vasopressors, intravenous fluids, and continuous replacement therapy. Outcome: Discharged home on day 11

Five patients were <65 years old and five patients were >65 years of age, with the youngest being 48 years old with a female predominance. Whether CKD was preexistent or not, the majority of the cases presented with acute vomiting and diarrhea, which caused pre-renal acute kidney injury (AKI) and precipitated the development of MALA. One hundred percent of the cases were discharged from the hospital, but discharge destinations are unavailable for four cases, as is data on rehospitalizations or follow-up. One patient was a nursing home resident. Five patients were discharged home, and of the four with unknown destinations, two of them were over age 70. Around 41% of patients never return home after discharge from the hospital to a nursing facility, in part due to physical debility and permanent cognitive deficits after acute illness [18]. It is important to recognize the risk factors of MALA in the outpatient and nursing home setting and prevent hospitalizations as some patients might become permanently debilitated from a long hospitalization. 

This case highlights the potential risks associated with metformin use in older adults with or without CKD. More importantly, it highlights the fact that sudden development of AKI from infections, dehydration, or decreasing oral intake due to acute illness, aging, or dementia is a huge risk factor for developing MALA. A study has shown nephrosclerosis in 2.7% of patients less than 30 years old, 58% in the age group of 60-69 years old, and 73% in those over 70 years old [9]. Cellular dysfunction and nephrosclerosis are expected physiological changes in the aging kidney that can cause sudden kidney injury in older adults causing their eGFR to drop acutely [19]. Age-related changes in renal function and decreased clearance of drugs place older adults at higher risk of developing MALA.

It is of importance to carefully monitor kidney function in patients taking metformin and either deprescribe or discontinue it completely based on eGFR since metformin is renally cleared and can accumulate if there is decreased renal clearance. Patients also need to be counselled on discontinuing or reducing metformin in acute illness. While there are guidelines on eGFR contraindications to metformin, there are no guidelines on metformin reduction or deprescribing in elderly patients who are at high risk of sudden AKI from sudden acute illness.

## Conclusions

Metformin use in older adults poses a risk whether preexistent CKD exists or not. Any AKI that adults are susceptible to very quickly as evidenced by the various case reports and discussion can precipitate MALA from regular metformin use. Anticipated physiological changes in the aging kidney, including cellular dysfunction and nephrosclerosis, can lead to AKI in older adults, causing a sudden drop in their eGFR. AKI causes decreased drug clearance placing older adults at a higher risk of developing MALA. Patients taking metformin can be counselled on reducing or discontinuing metformin during acute illness or states in which they have poor oral intake; however, no guidelines are available currently. More research is required to come up with guidelines on whether reducing or deprescribing metformin earlier would prevent morbidity, mortality, and adverse outcomes in older patients with diabetes. 
